# A rapid and simple method for routine determination of antibiotic sensitivity to biofilm populations of *Pseudomonas aeruginosa*

**DOI:** 10.1186/s12941-020-00350-6

**Published:** 2020-03-13

**Authors:** Dhammika Leshan Wannigama, Cameron Hurst, Parichart Hongsing, Lachlan Pearson, Thammakorn Saethang, Naphat Chantaravisoot, Uthaibhorn Singkham-in, Sirirat Luk-in, Robin James Storer, Tanittha Chatsuwan

**Affiliations:** 1 Department of Microbiology, Faculty of Medicine, King Chulalongkorn Memorial Hospital, Chulalongkorn University, Bangkok, Thailand; 2 School of Medicine, Faculty of Health and Medical Sciences, The University of Western Australia, Nedlands, WA Australia; 3 Department of Statistics, QIMR Berghofer Medical Research Institute, Brisbane, QLD Australia; 4 Center of Excellence in Biostatistics, Faculty of Medicine, Chulalongkorn University, Bangkok, Thailand; 5 School of Integrative Medicine, Mae Fah Luang University, Chiang Rai, Thailand; 6 Center of Excellence in Systems Biology, Research Affairs, Faculty of Medicine, Chulalongkorn University, Bangkok, Thailand; 7 Department of Biochemistry, Faculty of Medicine, Chulalongkorn University, Bangkok, Thailand; 8 Centre for Heart Research, Westmead Institute for Medical Research, Sydney, NSW Australia; 9 Department of Clinical Microbiology and Applied Technology, Faculty of Medical Technology, Mahidol University, Bangkok, Thailand; 10 Office of Research Affairs, Faculty of Medicine, Chulalongkorn University, Bangkok, Thailand; 11 Antimicrobial Resistance and Stewardship Research Unit, Faculty of Medicine, Chulalongkorn University, Bangkok, Thailand; 12 Department of Computer Science, Faculty of Science, Kasetsart University, Bangkok, Thailand

**Keywords:** Biofilms, *Pseudomonas aeruginosa*, Biofilm infections, Antibiofilm, Chronic bacterial infections, Antimicrobial susceptibility

## Abstract

Treatment of infections by *Pseudomonas aeruginosa* forming biofilms after antimicrobial testing on planktonic bacteria can result in substantial failure. Therefore, we offer a robust and simple experimental platform to test the impact of antimicrobials on biofilms. Antibiotic response patterns varied uniquely within biofilm formation capacity and minimal biofilm eradication concentrations (MBECs) has a significantly better discriminatory power than minimum inhibitory concentrations (MICs) to differentiate the overall efficiency of antibiotics to eradicate biofilm. Our resazurin-based 96-well-plate platform is able to emulate bacterial responses to antibiotics under biofilm conditions in a fast, simple, and cost-effective screening method adaptable to automation, and warrants trials in the clinic.

## Introduction

The properties of bacteria in biofilms differ from those of planktonic bacteria [1, 2], and bacteria in biofilms have extreme tolerance to immune responses and antimicrobial therapy [3, 4]. Biofilm formation is therefore an obstacle to the treatment of chronic infections with *Pseudomonas aeruginosa*, most of which are associated with biofilms [1, 2]. Despite the negative impact of biofilms, to our knowledge, no treatment that directly targets bacteria in biofilms has yet been developed [1, 5].

Biofilm recalcitrance to antibiotics is based on a mixture of resistance and tolerance [1, 6]. Clinical treatments with antibiotics are usually determined from minimum inhibitory concentrations (MICs) for planktonic bacteria, and, as a result, patients may suffer from persistent infection over the course of weeks, or even months, often with recurrence of even more aggressive exacerbations [1, 7]. Patients harboring bacteria within biofilms require higher doses of antibiotics and more prolonged courses of treatment than treatment suggested by testing with planktonic bacteria [1, 8].

Patients with chronic infections treated with antibiotic regimens based on biofilm susceptibility-testing have better clinical outcomes than those treated with regimens based on methods measuring susceptibility to planktonic bacteria [5, 9].

In a previous study, we developed a simplified antibiotic susceptibility assay based on a standardized model to quantify viable cells in biofilms of *Acinetobacter baumannii* [10, 11]. Our assay is based on the quantitative measurement of metabolically active cells using PrestoBlue, a resazurin (7-hydroxy-3H-phenoxazin-3-one-10-oxide)-based viability indicator. The results clearly demonstrated the significant discriminatory power of the assay (MBEC) to differentiate antibiotic efficacy on biofilms compared with current MIC-based assays [10, 11]. While the new assay has proven to present an effective model of biofilm formation, in this article we describe its reproducibility and applicability for rapid antibiotic susceptibility testing of *P. aeruginosa* biofilms in a clinical laboratory setting.

## Materials and methods

### Strains and culture conditions

Clinical isolates used in this study were selected from a *P. aeruginosa* strain repository in the Department of Microbiology, King Chulalongkorn Memorial Hospital, Bangkok, Thailand. The strains were stored at the repository collection after standard characterization and identification, including 16S rRNA sequencing as described previously [12] (Additional file 1). Clinical strains were isolated during 2016–2017 from chronically-infected patients as part of their standard care. The *P. aeruginosa* clinical isolates were cultured on Müller-Hinton agar plates at 37 °C. Without preference, we selected 137 unduplicated clinical isolates representing 137 patients and 14 collection sites with relevant chronic infection (including urine, bile, corneal scrapings, nasal swabs, tissue, blood, device related, broncho–alveolar aspirates, ear swabs, eye swabs, conjunctival swabs, wound pus, endotracheal aspirates, and sputum). Strains from patients with multiple sites of infection were excluded, and we only included samples from patients with infection at a single site. All isolates were stored at − 80 °C in tryptic soy broth with 15% glycerol until used in subsequent experiments.

### Antibiotics and agents

The biofilm eradication activity of 7 antibiotics was tested against the subset of clinical isolates (n = 137). Gentamicin, amikacin, ciprofloxacin, meropenem, colistin, and ceftazidime were all from Sigma-Aldrich. Susceptibility testing for fosfomycin (Wako Chemicals) was determined by supplementation with 25 μg/mL glucose-6-phosphate. Antibiotic stock solutions were prepared less than 24 h before use. Antibiotics were dissolved in cation-adjusted Müller-Hinton II broth (MHIIB) (Becton Dickinson) medium and sterilized by filtration through a membrane (0.22 μm pores). Serial dilutions of the stocks were prepared in MHIIB immediately before use.

### Testing susceptibility to antibiotics

The planktonic MIC were established using standard techniques according to European Committee on Antimicrobial Susceptibility Testing (EUCAST) criteria [13] and Clinical and Laboratory Standards Institute (CLSI) guidelines [14]. *Escherichia coli* ATCC 25922, and *P. aeruginosa* ATCC 27853 were used as quality control strains. Minimal biofilm eradication concentrations (MBEC) were established using our previously develop fluorometric-based assay to calculate the number of viable cells within the biofilm as described previously [10]. In brief, MBECs were determined by adding the serially diluted antibiotics to mature biofilms and incubating at 37 °C for 24 h before staining with PrestoBlue. Before adding the antibiotics, any nonadherent cells were removed from the mature biofilms by 3 gentle washes with MHIIB. Cell viability was calculated using the following formula: cell viability (%) = ((mean signal of corresponding well − mean signal of negative control well)/(mean signal of positive control well − mean signal of negative control well)) × 100. Two cut-off values (50% and 75% nonviable cells) were used to determine the MBEC. All experiments were performed in triplicate and repeated 3 times. As a comparison we also used the 96‐well Calgary Biofilm Device (CBD) (Innovotech, Calgary, Canada) as described previously to determine MBEC [15].

### Biofilm formation quantification and classification

Two methods were used to quantify [16] and classify [17] the biofilm structure by Crystal Violet staining followed by confocal laser scanning microscopy using live or dead bacterial staining as described previously [18]. Mean absorbances and their standard deviations (SDs) were calculated for all tested strains and negative controls, determined in triplicate and repeated 3 times. The clinical isolates were classified as described previously [17].

### Statistical analyses

Continuous variables are summarized using means and SDs, and categorical variables as counts and percentages. Levels of *P. aeruginosa* drug susceptibility are represented in 2 ways: a continuous measure of concentration; and an ordinal categorical form representing biofilm formation (negative, weak, moderate, or strong); both of these outcomes were measured repeatedly over time for each isolate. Linear mixed modeling was used to compare concentrations between test types (MIC vs. MBEC) over time. We then examined which test types (MIC vs. MBEC) were more successful in allowing concentration to be used to distinguish between biofilm formations (negative, weak, moderate, or strong) using ordinal logistic mixed effects regression. Finally, we examined whether concentration could be used to predict biofilm formation using multinomial logistic regression. All analysis was conducted using the R statistical package [19], linear mixed modeling was performed using the R library, lme4 [20], and ordinal logistic mixed effect modeling using the R library, ordinal [21], and multinomial logistic regression using the R library, nnet [22]. *P* < 0.05 was considered significant for all inferential analysis.

## Results

### Association between antibiotic resistance and biofilm formation

**Fig. 1:**
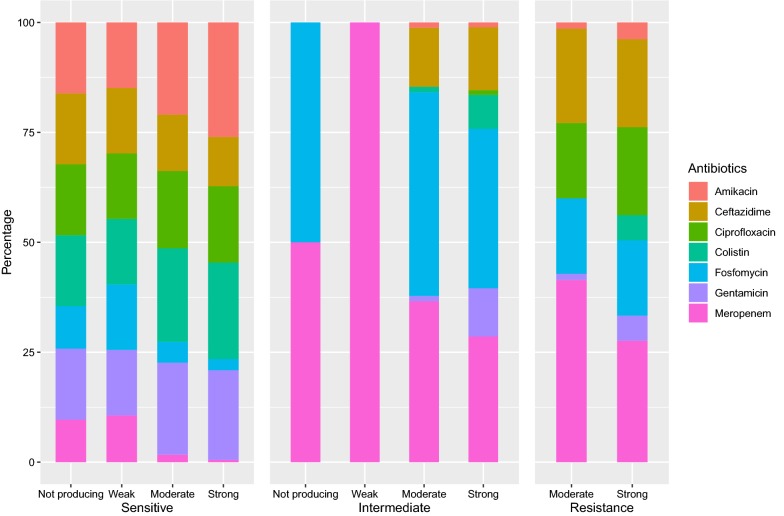
Antibiotic susceptibility of clinical isolates of *P. aeruginosa* to 7 antibiotics among various biofilm production capacities as a percentage

The planktonic antibiotic resistance profile of each isolate revealed that resistance to meropenem was most common, followed in order by ceftazidime, ciprofloxacin, and fosfomycin (Fig. 1). Most strains showed high susceptibility to colistin, amikacin, and gentamicin. In total, 127 (92%) isolates were positive for biofilm formation, and 56 (46%) isolates formed a stronger biofilm. No significant difference was found in terms of biovolume between the fluorometric assay and the Calgary Biofilm Device (*P* = 1.0092; Additional file 2). The composition of the biofilm formation categories with respect to resistance profile showed that antibiotic resistant isolates form stronger biofilms than sensitive isolates (*P* < 0.001; Fig. 1). Strong and moderate biofilms showed similar levels of enhancement in all 3 antibiotic assessment groups.

### Correlation between biofilm formation and susceptibility test type

**Fig. 2:**
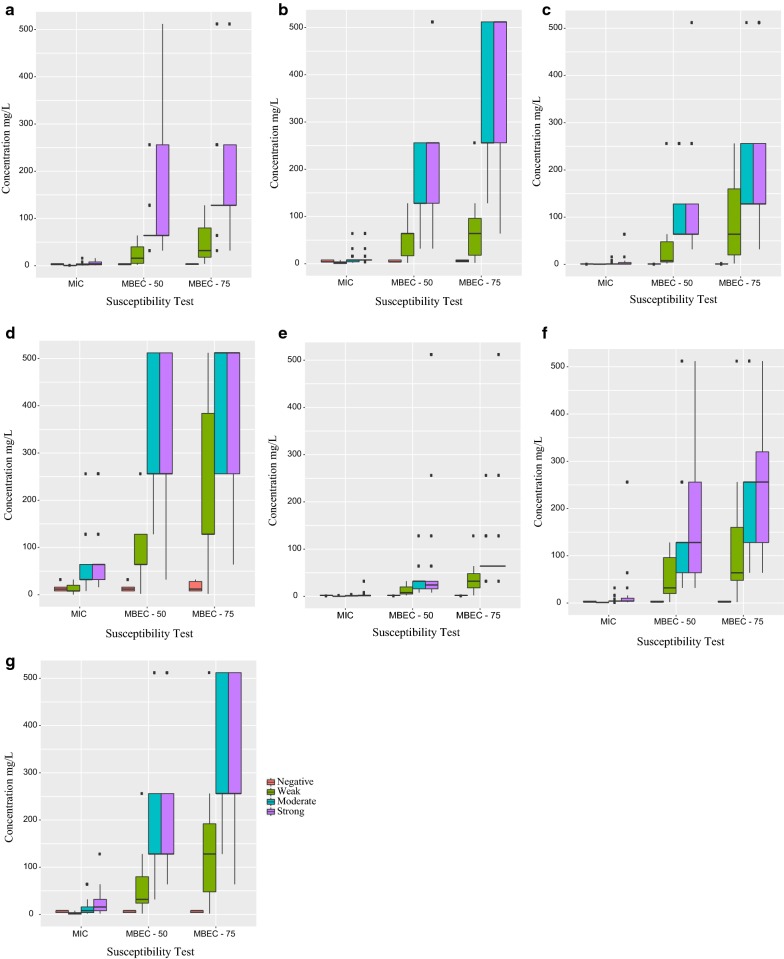
Association between the level of biofilm formation (negative, weak, moderate, or strong) and susceptibility test types to 7 antibiotics for *P. aeruginosa* clinical isolates. **a** gentamicin, **b** amikacin, **c** ciprofloxacin, **d** meropenem, **e** colistin, **f** fosfomycin, and **g** ceftazidime. *MIC* minimum inhibitory concentration of planktonic cells based on conventional susceptibility test, *MBEC* minimum biofilm eradication concentration based on PrestoBlue cell viability indicator

To determine whether biofilm formation is correlated with susceptibility test type, we compared the biofilm forming capacities between strains with 3 types of tests for each of the 7 antibiotics. We found that an overall MBEC susceptibility test significantly modifies the relationship between biofilm formation and antibiotic concentration (*P* < 0.001; Fig. 2). Strong and moderate biofilms likely exhibit similar trends for all of the antibiotics tested. The trend is very pronounced for amikacin and fosfomycin (MBEC-75 > MBEC-50 > MIC). Variation of the strong and moderate biofilm in MBEC-75 is much more pronounced for amikacin, meropenem, and ceftazidime than other antibiotics, particularly colistin, where variation was comparatively low. MIC tests did not show any differences in association with weak, moderate, or strong biofilms.

### Relationship between susceptibility test types and antibiotics

**Fig. 3:**
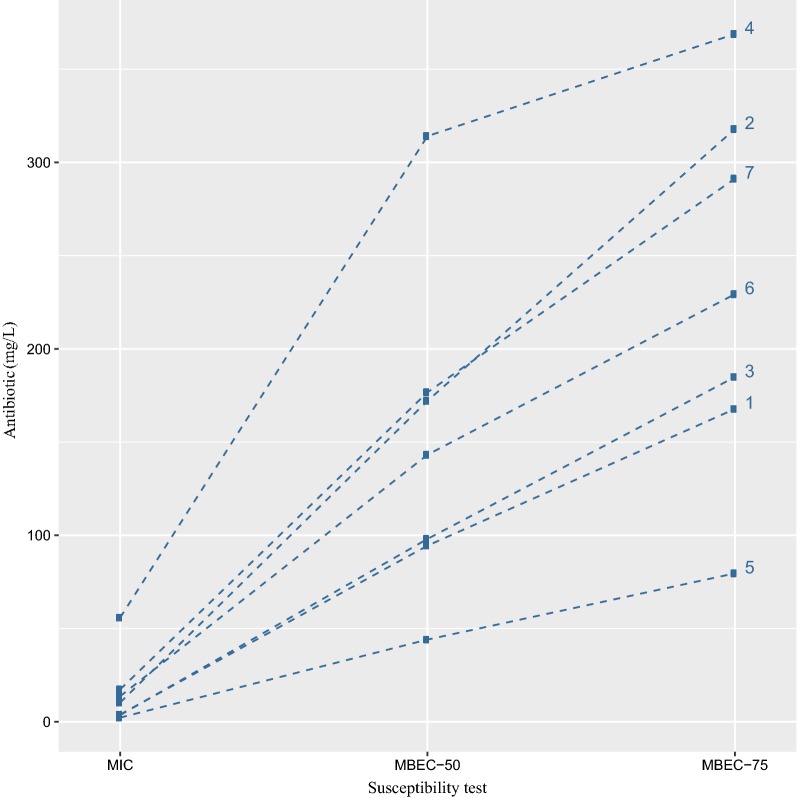
Relationship between susceptibility tests and 7 antibiotics for *P. aeruginosa* clinical isolates: (1) gentamicin, (2) amikacin, (3) ciprofloxacin, (4) meropenem, (5) colistin, (6) fosfomycin, and (7) ceftazidime. *MIC* minimum inhibitory concentration of planktonic cells based on conventional susceptibility test, *MBEC* minimum biofilm eradication concentration based on PrestoBlue cell viability indicator

A linear mixed model revealed a significant relationship between the type of susceptibility test and antibiotics (*Z*_LRT_^2^ = 312.26, 12 df, *P* < 0.001) showing that the magnitude of differences between tests was modified by antibiotics. Figure 3 shows all antibiotics except meropenem and colistin tended to have the same general pattern (MBEC-75 > MBEC-50 > MIC). Whereas with meropenem, the difference between MIC and MBEC-50 is much more pronounced, and for colistin the difference is much less pronounced. No significant difference was found in terms of MBEC between the fluorometric assay and the Calgary Biofilm Device (*P* = 0.998; Table 1).

**Table 1:** Susceptibility range for each of the antibiotics based on planktonic population (MIC) and as a biofilm population (MBEC) derived by the fluorometric-based assay and Calgary Biofilm Device

Antimicrobial agents	Broth microdilution	Fluorometric-based assay		Calgary Biofilm Device
	MIC^a^	MBEC-50^b^	MBEC-75^b^	MBEC
Gentamicin	0.25–16	2–128	4–512	2–512
Amikacin	0.25–64	2–256	2–512	2–512
Ciprofloxacin	0.25–64	0.5–128	1–512	0.5–512
Meropenem	0.25–64	2–256	2–512	2–512
Colistin	0.25–32	1–128	1–256	1–256
Fosfomycin	0.25–256	2–512	2–512	2–512
Ceftazidime	0.25–128	2–512	2–512	2–512

^a^Minimal inhibitory concentrations (MIC, mg mL^−1^) of planktonic cells

^b^Minimal biofilm eradication concentrations (MBEC, mg mL^−1^) were categorized as responsive reaching about 50% and 75% of the total nonviable cells within a given antibiotic concentration range

### Association of susceptibility test types, biofilm formation, and antibiotic concentrations

The associations between odd ratios of MIC, MBEC (50 and 75) and concentration attribute of each antibiotic are shown in Table 2. It is important to note that for this analysis we employed standardized concentrations (*Z*-scores) to avoid higher (raw) values of concentrations making associations appear more trivial. For each antibiotic, the odds ratios from MBEC-50 and 75 tests are a reflection of the higher level of associations than with MIC, except for fosfomycin. Notably, for gentamicin and amikacin the odds ratio of MBEC-50 was higher than MBEC-75, but both displayed a similar level of significance between MBEC-50 and 75. However, for colistin, a similar level of significance was observed for the association between MIC and MBEC-50.

**Table 2:** Odds ratios with 95% CIs from ordinal mixed effect regression by susceptibility test types for each of the antibiotics based on standardized (Z-score) concentrations

Antimicrobial agents	MIC^a^		MBEC-50^b^		MBEC-75^c^	
	OR_z_ (95% CIs)^d^	BFCA (%)^e^	OR_z_ (95% CIs)^d^	BFCA (%)^e^	OR_z_ (95% CIs)^d^	BFCA (%)^e^
Gentamicin	2.31 (0.00132, 0.00406)**	61	11.05 (0.00390, 0.03132)***	65	4.18 (0.00227, 0.00769)***	62
Amikacin	1.75 (0.00108, 0.00284)*	50	5.00 (0.00251, 0.00996)***	64	4.06 (0.00244, 0.00676)***	64
Ciprofloxacin	2.40 (0.00077, 0.00751)	51	3.57 (0.00196, 0.00650)***	58	5.40 (0.00279, 0.01047)***	55
Meropenem	2.49 (0.00135, 0.00459)**	50	2.99 (0.00196, 0.00456)***	58	2.62 (0.00173, 0.00396)***	54
Colistin	8.66 (0.00153, 0.048.96)*	54	7.49 (0.00154, 0.03636)*	58	18.66 (0.00427, 0.08148)***	58
Fosfomycin	61.10 (0.00149, 2.50718)*	54	2.91 (0.00170, 0.00499)***	58	2.84 (0.00182, 0.00444)***	58
Ceftazidime	1.54 (0.00103, 0.00231)*	53	2.20 (0.00145, 0.00333)***	56	3.53 (0.00216, 0.00576)***	55

*P < 0.05; **P < 0.01; ***P < 0.001

^a^Minimal inhibitory concentrations (MIC, mg mL^−1^) of planktonic cells

^b^Minimal biofilm eradication concentrations (MBEC, mg mL^−1^) were categorized as responsive reaching about 50% and 75% of the total nonviable cells within a given antibiotic concentration range

^c^Odds ratio with 95% confidence interval

^d^Biofilm formation classification accuracy (negative, weak, moderate, or strong)

For all the strains tested, the accuracy of biofilm classification was higher for both MBEC-50 and 75 tests compared with a MIC test for each antibiotic. We can see that concentrations using MBEC-50 correctly predicted the biofilm formation in gentamicin, ciprofloxacin, meropenem, and fosfomycin, followed by ceftazidime. MBEC-75 is able to predict biofilm formation for colistin with 58% accuracy, and for amikacin both MBEC-50 and 75 displayed similar levels.

## Discussion

Current guidelines or antibiotic therapies, based on planktonic bacteria are often unable to offer a successful path to the treatment of biofilm infections [1, 6]. As biofilm bacteria are inherently more tolerant to antibiotics, it is necessary to determine biofilm specific antibiotic susceptibility to predict therapeutic success.

Therefore, the key advantages of the present assay are: first, that it simplifies the steps of biofilm formation and is able to produce biofilms equivalent to those produced by the Calgary Biofilm Device, making it standard assay system compliant with antibiotic susceptibility testing for biofilm infections. The reproducibility of the results for biofilms formed on each well of the 96-well plate and reproducible biofilm categories (weak, moderate, or strong) attributed to each clinical isolate demonstrate that this fluorometric assay can produce biofilms equivalent to those of each peg of the Calgary Biofilm Device lid. It is therefore possible to provide equivalent and clinically relevant biofilm that can be exposed to multiple antibiotics in a single 96-well plate with viability assessment to provide accurate antibiotic selection in a clinically useful time frame. The assay requires no specific peg lid plates or changing bottom-well plates at each step, making the process much simpler to set up than the Calgary Biofilm Device, and thus eliminates possible contamination and reduces expense. The use of a single 96-well plate for each testing step greatly reduces the time required to determine the antibiotic susceptibilities of biofilms and minimized the workload. The fluorometric-based assay is also amenable to automation because it is based on typical standard 96-well plates.

Second, the assay is a valid way to differentiate antibiofilm effectiveness based on biofilm formation capacity by resembling clinical situations. We observed marked differences between MBEC concentration patterns of each tested antibiotics to weak, moderate, and strong biofilms. Some antibiotics are able to penetrate moderate biofilms readily, but strong biofilms poorly. This is an interesting observation, in that such differences may also reflect more fundamental differences in the biological characteristics of biofilm structure [4, 23], and metabolic or physiological factors [1, 4, 24] of clinical isolates that are not accounted for in MIC testing. Third, a clear difference in antibiotic susceptibility was seen between planktonic populations of each of the isolates tested and the biofilm populations of the same isolate. Each of the isolates had a unique biofilm susceptibility to the each of antibiotics tested. The biofilms of clinical isolates  of *P. aeruginosa* proved to be very difficult to eradicate, with only colistin being effective at achievable drug concentrations, with the other aminoglycoside tested (gentamicin), and ciprofloxacin showing just some activity against weak biofilms. Indeed, the higher levels of significant odds ratios with biofilm formation classification accuracy of MBEC-50 and 75 tests suggest that they have better discriminatory power than an MIC test. The accuracy of biofilm formation classification reflects that, to overcome the 50% cell death in a biofilm is crucial for the efficacy of particular antibiotics. The manner in which biofilm-induced tolerance and intrinsic resistance become integrated to promote biofilm-specific antibiotic resistance was shown. These data could be interpreted to indicate that the MIC is predictive of antibiotic efficacy against planktonic bacterial cells, but not for those living within biofilms. This is consistent with what is often seen as symptomatic relief by eliminating the planktonic population [1, 6, 25]. However, because the biofilm is not eliminated by antibiotic treatment, reinfection occurs once the antibiotic is removed [1, 6, 25].

The present work is limited by the following considerations. In the presently described assay, the effect of antibiotics on biofilm were determined, although appropriate standard reference values required to clear infections in vivo remain unclear. Combining the present quantitative screening of bacterial biofilm-specific antibiotic resistance with clinical trials of antibiotics would clarify the clinical applicability of the assay.

In conclusion, the presently described quantitative screening assay of bacterial biofilm-specific antibiotic resistance assay is a versatile, easy to manage, and robust method that should help to improve treatment of infections that are threats in the clinic.

## Supplementary information

**Additional file 1.** Biofilm formation and Testing susceptibility to antibiotics.

**Additional file 2: Figure S1.** Distribution of clinical isolates (n = 137) of *P. aeruginosa* biovolume within the fluorometric-based assay and Calgary Biofilm Device.

## Data Availability

The authors confirm that the data supporting the findings of this study are available within the article and its Additional information.

## Abbreviations

ATCC: American Type Culture Collection
CLSI: Clinical and Laboratory Standards Institute
EUCAST: European Committee on Antimicrobial Susceptibility Testing
MHIIB: Müller-Hinton II broth
MBEC: Minimum biofilm eradication concentration
MIC: Minimum inhibitory concentration
MBEC-75: 75% Nonviable cells
MBEC-50: 50% Nonviable cells
OD: Optical density
SD: Standard deviations
