# Supplementary material for: A rapid and simple method for routine determination of antibiotic sensitivity to biofilm populations of Pseudomonas aeruginosa
Source: Ann Clin Microbiol Antimicrob. 2020 Mar 13;19:8. doi: 10.1186/s12941-020-00350-6 (PMC7071750; doi:10.1186/s12941-020-00350-6)
Supplement: Supplementary file 1 — Additional file 1. Biofilm formation and Testing susceptibility to antibiotics. [file 12941_2020_350_MOESM1_ESM.docx]

Short report

**A rapid and simple method for routine determination of antibiotic sensitivity to biofilm populations of *Pseudomonas aeruginosa***

Dhammika Leshan Wannigama^1,2,11^, Cameron Hurst^3,4^, Parichart Hongsing^5^, Lachlan Pearson^6,8^, Thammakorn Saethang^6,12^, Naphat Chantaravisoot ^6,7^, Uthaibhorn Singkham-in^1,11^, Sirirat Luk-in^1,9,11^, Robin James Storer^10^, Tanittha Chatsuwan^1,11,*^

^1^Department of Microbiology, Faculty of Medicine, Chulalongkorn University, King Chulalongkorn Memorial Hospital, Bangkok, Thailand

^2^School of Medicine, Faculty of Health and Medical Sciences, The University of Western Australia, Nedlands, Western Australia, Australia.

^3^Department of Statistics, QIMR Berghofer Medical Research Institute, Brisbane, Queensland, Australia.

^4^Center of Excellence in Biostatistics, Faculty of Medicine, Chulalongkorn University, Bangkok, Thailand.

^5^ School of Integrative Medicine of Mae Fah Luang University, Chiang Rai, Thailand.

^6^ Center of Excellence in Systems Biology, Research Affairs, Faculty of Medicine, Chulalongkorn University, Bangkok, Thailand.

^7^ Department of Biochemistry, Faculty of Medicine, Chulalongkorn University, Bangkok, Thailand.

^8^ Centre for Heart Research, Westmead Institute for Medical Research, Sydney, New South Wales, Australia.

^9^ Department of Clinical Microbiology and Applied Technology, Faculty of Medical Technology, Mahidol University, Bangkok, Thailand.

^10^ Office of Research Affairs, Faculty of Medicine, Chulalongkorn University, Bangkok, Thailand

^11^ Antimicrobial Resistance and Stewardship Research Unit, Faculty of Medicine, Chulalongkorn University, Bangkok, Thailand.

^12^ Department of Computer Science, Faculty of Science, Kasetsart University, Bangkok, Thailand.

**Additional information**

**Additional file 1. Materials and methods**

**Biofilm formation**

Initially, a pure culture of a single colony of *P. aeruginosa* was inoculated into 2 mL of Müller–Hinton II broth (MHIIB) medium in a tube and incubated in an orbital shaker (200 rpm) at 37°C for about 16 h. Subsequently, a subculture was prepared from the incubated culture by diluting it with fresh MHIIB to an optical density (OD) of 0.02 at 600 nm (5 × 10^7^ CFU/mL) and 100 μL aliquots were added in triplicate to flat-bottomed 96-well polystyrene microtitre plates (SPL Life Sciences), using uninoculated MHIIB (100 μL) added to the same plate in triplicate as a negative control, the plates were incubated at 37°C for 24 h. After standardizing the conditions, we used the procedure to test the 117 biofilm-positive, and 20 biofilm-negative clinical isolates for their antimicrobial susceptibility profile under biofilm growth conditions. All experiments were performed in triplicate and repeated three times.

**Testing susceptibility to antibiotics**

Minimal biofilm eradication concentrations (MBEC) were established by adding serially diluted antibiotics to mature biofilms and incubating them together at 37°C for 24 h, with subsequent staining by PrestoBlue. Before adding the antibiotics, any nonadherent cells were removed from the mature biofilms by three gentle washes with MHIIB. Cell viability was calculated using the following formula: cell viability (%) = ((mean signal of corresponding well – mean signal of negative control well)/(mean signal of positive control well – mean signal of negative control well)) × 100. Two cut-off values (50% and 75% nonviable cells) were used to determine the MBEC. All experiments were performed in triplicate and repeated three times.

**Supplementary Table 1**. 16S rDNA-based primer set and conditions used to identify *P. aeruginosa* in the clinical isolates (reference 12).

| **Primer** | **Sequence (5′–3′)** | **Target** | **Annealing temp (°C)** | **Location** | **Product size (bp)** |
| --- | --- | --- | --- | --- | --- |
| PA-SS-F | GGGGGATCTTCGGACCTCA | *P. aeruginosa* | 58 | 189–206 | 956 |
| PA-SS-R | TCCTTAGAGTGCCCACCCG |  |  | 1124–1144 |  |
