# Supplementary material for: A rapid and simple method for routine determination of antibiotic sensitivity to biofilm populations of Pseudomonas aeruginosa
Source: Ann Clin Microbiol Antimicrob. 2020 Mar 13;19:8. doi: 10.1186/s12941-020-00350-6 (PMC7071750; doi:10.1186/s12941-020-00350-6)
Supplement: Supplementary file 2 — Additional file 2: Figure S1. Distribution of clinical isolates (n = 137) of P. aeruginosa biovolume within the fluorometric-based assay and Calgary Biofilm Device. [file 12941_2020_350_MOESM2_ESM.pdf]

Short report

A rapid and simple method for routine determination of antibiotic sensitivity to biofilm populations of *Pseudomonas aeruginosa*

Dhammika Leshan Wannigama[1,2,11], Cameron Hurst[3,4], Parichart Hongsing[5], Lachlan Pearson[6,8], Thammakorn Saethang[6,12], Naphat Chantaravisoot [6,7], Uthaibhorn Singkham-in[1,11], Sirirat Luk-in[1,9,11], Robin James Storer[10], Tanittha Chatsuwan[1,11,*]

[1]Department of Microbiology, Faculty of Medicine, Chulalongkorn University, King Chulalongkorn Memorial Hospital, Bangkok, Thailand

[2]School of Medicine, Faculty of Health and Medical Sciences, The University of Western Australia, Nedlands, Western Australia, Australia.

[3]Department of Statistics, QIMR Berghofer Medical Research Institute, Brisbane, Queensland, Australia.

[4]Center of Excellence in Biostatistics, Faculty of Medicine, Chulalongkorn University, Bangkok, Thailand.

[5] School of Integrative Medicine of Mae Fah Luang University, Chiang Rai, Thailand.

[6] Center of Excellence in Systems Biology, Research Affairs, Faculty of Medicine, Chulalongkorn University, Bangkok, Thailand.

[7] Department of Biochemistry, Faculty of Medicine, Chulalongkorn University, Bangkok, Thailand.

[8] Centre for Heart Research, Westmead Institute for Medical Research, Sydney, New South

Wales, Australia.

[9] Department of Clinical Microbiology and Applied Technology, Faculty of Medical Technology, Mahidol University, Bangkok, Thailand.

[10] Office of Research Affairs, Faculty of Medicine, Chulalongkorn University, Bangkok, Thailand

[11] Antimicrobial Resistance and Stewardship Research Unit, Faculty of Medicine, Chulalongkorn University, Bangkok, Thailand.

[12] Department of Computer Science, Faculty of Science, Kasetsart University, Bangkok, Thailand.

Additional information

Additional file 2: Figure S1 Distribution of clinical isolates (n = 137) of *P. aeruginosa* biovolume within the fluorometric-based assay and Calgary Biofilm Device. Biovolume data is based on mean values of three independent replicates of z-stack measurements by confocal laser scanning microscopy.

Figure S1

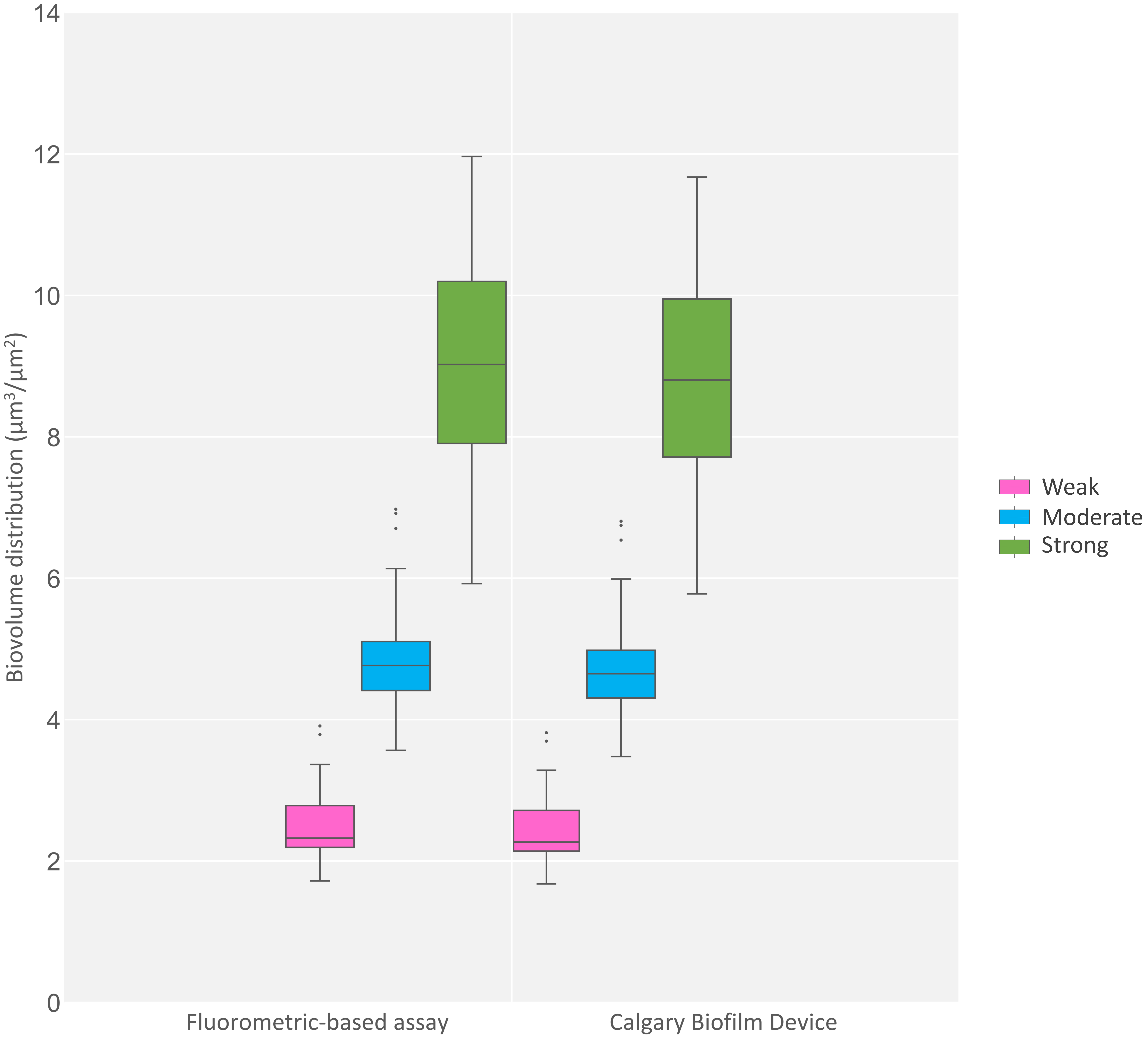

14
12
10
8
6
4
2
0
Biovolume distribution (μm³/μm²)
Fluorometric-based assay
Calgary Biofilm Device
Weak
Moderate
Strong
